# Single-Step Deposition of Chalcopyrite (CuFeS_2_) Thin Films

**DOI:** 10.1021/acsomega.4c08647

**Published:** 2024-12-04

**Authors:** Valmar da Silva Severiano Sobrinho, Thercio Henrique de Carvalho Costa, Michelle Cequeira Feitor, Maxwell Santana Libório, Rômulo Ribeiro Magalhães de Sousa, Álvaro Albueno da Silva Linhares, Pâmala Samara Vieira, Luciano Lucas Fernandes Lima, Cleânio da Luz Lima, Edcleide Maria Araújo

**Affiliations:** † Materials Science and Engineering Post-Graduation − Federal University of Campina Grande (UFCG), Campina Grande 58429-900, PB, Brazil; ‡ Mechanical Engineering Post-Graduation − Federal University of Rio Grande do Norte (UFRN), Natal 59078-970, RN, Brazil; § Science and Technology School − Federal University of Rio Grande do Norte (UFRN), Natal 59078-970, RN, Brazil; ∥ Mechanical Department – Federal University of Piauí (UFPI), Teresina 64049-550, PI, Brazil; ⊥ Materials Science and Engineering Post-Graduation − Federal University of Rio Grande do Norte (UFRN), Natal 59078-970, RN, Brazil; # Physic Post-Graduation − Federal University of Piauí (UFPI), Teresina 64049-550, PI, Brazil

## Abstract

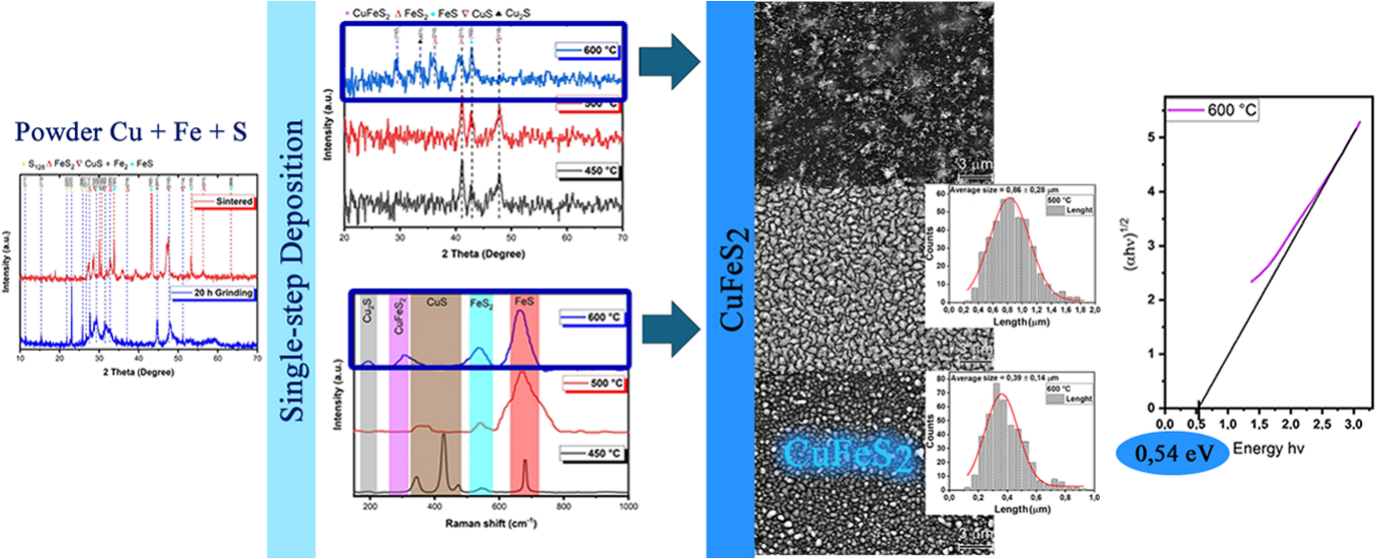

Thin films of chalcopyrite, CuFeS_2_, are promising candidates for use as absorber layers in photovoltaic cells due to their low band gap and high absorbance. These films are typically deposited in two or three steps, always involving an annealing process. In this work, the CuFeS_2_ film was deposited on a glass substrate in a single deposition step using the cathodic cylindrical plasma deposition (CCyPD) technique. The film samples deposited were analyzed by X-ray diffraction (XRD) and Raman spectroscopy, the film thickness was measured using the optical method, and FEG-SEM analyzed the surface structural morphology. The results showed a strong dependence on the deposition temperature for phase formation, with chalcopyrite being obtained for films deposited at 600 °C. At this temperature, a uniformly distributed film with uniform grain sizes was obtained, and the experimentally obtained band gap values of the films were consistent with the theoretical values reported in the literature, demonstrating the technique’s effectiveness and precision in producing high-quality films.

## Introduction

Chalcopyrite is theoretically and experimentally studied as a promising material for electronic applications, such as light-emitting diodes, photocatalytic cells, and nonlinear optical devices. Among chalcopyrites, those of the ABX_2_ type (A = Cu, Al, Ag, Nb, Na; B = Cr, Al, Fe, B, In, Ga; X = S, Se, Te) have gained prominence due to their high stability and good electronic properties.^1−8^

Thin chalcopyrite films derived from Se, In, and Te possess good thermoelectric properties. Still, they are derived from toxic or rare materials, making sulfide-based chalcopyrites a more cost-effective option for energy applications. Among these, CuFeS_2_, which has a band gap of approximately 0.50 eV and a high absorption coefficient, is promising for use as an absorber layer in photovoltaic cells.^9−12^

Such films have already been successfully produced in the literature using two- and three-step processes. CuAl(SeTe)_2_ chalcopyrite films were produced by vacuum evaporation in Al/Cu/(SeTe) multilayers, followed by high-temperature annealing for the formation and crystallization of CuAl(SeTe)_2_.^13−17^

Several thin films of selenium- and sulfur-based chalcopyrite, such as Cu(InGaAlFe)(SeS)_2_, were deposited by evaporating successive layers of Cu/(InGaAlFe)/Cu··· (InGaAlFe)/Cu, with substrates kept at high temperatures, undergoing selenization or sulfurization processes in a Se- or S-rich atmosphere, followed by annealing, ultimately forming the desired chalcopyrite thin film.^18−27^

The cage cathodic cylinder deposition (CCyPD) method was described by de Medeiros Neto et al.^28^ This work offers a solution for obtaining chalcopyrite in a single deposition step. This work aimed to deposit a CuFeS_2_ chalcopyrite thin film in a single step using the CCyPD method. The influence of temperature on the formation of the desired phase was evaluated, and the films’ optical, electrical, and structural properties were studied.

## Materials and Methods

The raw materials used for the production of chalcopyrite films were iron powder (99.5%), copper (98%), and sulfur (99.5%). The films were deposited on soda-lime glass microscope slides with a rectangular geometry, transparent, and free of surface imperfections, measuring 26 mm × 76 mm with 1.0 and 1.2 mm thickness. The samples underwent a preparation process to reduce surface impurities and effectively prepare them for film deposition. They were washed with neutral detergent and then cleaned in an ultrasonic bath with acetone, ethanol, and deionized water for 30 min. Finally, the samples were dried with a nitrogen jet, as described by Sousa and da Cunha.^29^

The Fe, Cu, and S powders were weighed in the proportions of 30.5% Fe, 34.5% Cu, and 35.0% S, the natural proportions of chalcopyrite,^30^ and ground in a Pulverisette 6 (Fritsch) high-energy planetary mill for 20 h with a ratio of 3 g of grinding media for every 1 g of powder and a rotation speed of 400 rpm, using alcohol as a lubricant for better microstructural homogenization.^23^ After milling, the powder was placed in a drying and sterilization oven for 24 h at 70 °C. The resulting powder from this process was pressed with a load of 2 tons for 1 min to form the cathodic cylinders, which were then sintered in a plasma reactor for 2 h at 400 °C under a vacuum of 2 mbar and a controlled atmosphere of 8 sccm of Ar and 2 sccm of H_2_.

The produced cylinders were used for film deposition using the cathodic cylinder plasma deposition (CCyPD) technique described by de Medeiros Neto et al.^28^ This technique utilizes the same vacuum and atmosphere conditions used for sintering, varying the temperature at 450, 500, and 600 °C. The study included premixed powder after 20 h of milling, powder after sintering, and films deposited on glass. The deposition parameters are summarized in Table 1.

**Table 1:** Deposition Parameters

parameters	
working pressure [mBar]	2
deposition time [h]	6
argon flow [sccm]	8
hydrogen flow [sccm]	2
temperature [°C]	450, 500, and 600

The X-ray diffraction (XRD) analysis was performed using a Shimadzu XRD-7000 high-resolution diffractometer with copper Kα radiation, operating at 40 kV and 30 mA, with a grazing incidence of 3°, and a step size of 0.02° every 0.5 s. For the interpretation of the XRD data, relevant literature articles were consulted, and the crystallographic databases JCPDS (Joint Committee on Powder Diffraction Standards), ICDD (International Center for Diffraction Data), ICOD (International Crystallography Open Database), and ICCD (International Center for Crystallographic Data) were utilized.

Raman scattering was conducted using the Lab-RAM HR Evolution system (HORIBA Scientific) with 16 mW, a wavelength of 532 nm, a 10% power filter, an acquisition time of 10 s, and an accumulation of 10 measurements over a spectral range of 100–1000 cm^–1^. Transmittance was measured with a GENESYSTM 10S UV–vis spectrophotometer from THERMO FISHER SCIENTIFIC across a spectral range between 190 and 1100 nm with a scanning speed of 2.0 nm/s. Top morphological images of the deposited films were obtained by using a Zeiss Auriga 40 FEG-SEM, with a filament voltage of 5 kV and a working distance of 5.0 mm. The thickness of the films was calculated from transmittance data using the Pointwise Unconstrained Minimization Approach (PUMA) method.^31^ The band gap of the films was calculated using the Tauc linear extrapolation method.^32^

## Results and Discussion

**Figure 1:**
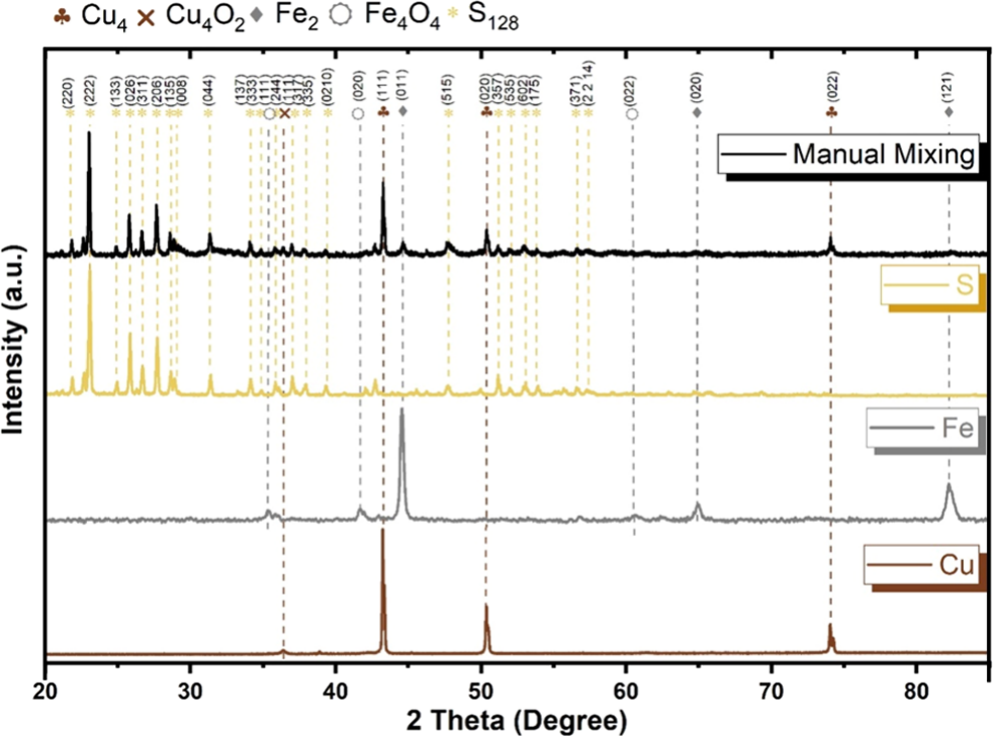
Pure Cu, Fe, and S powders and a manually mixed Cu–Fe–S mixture.

**Figure 2:**
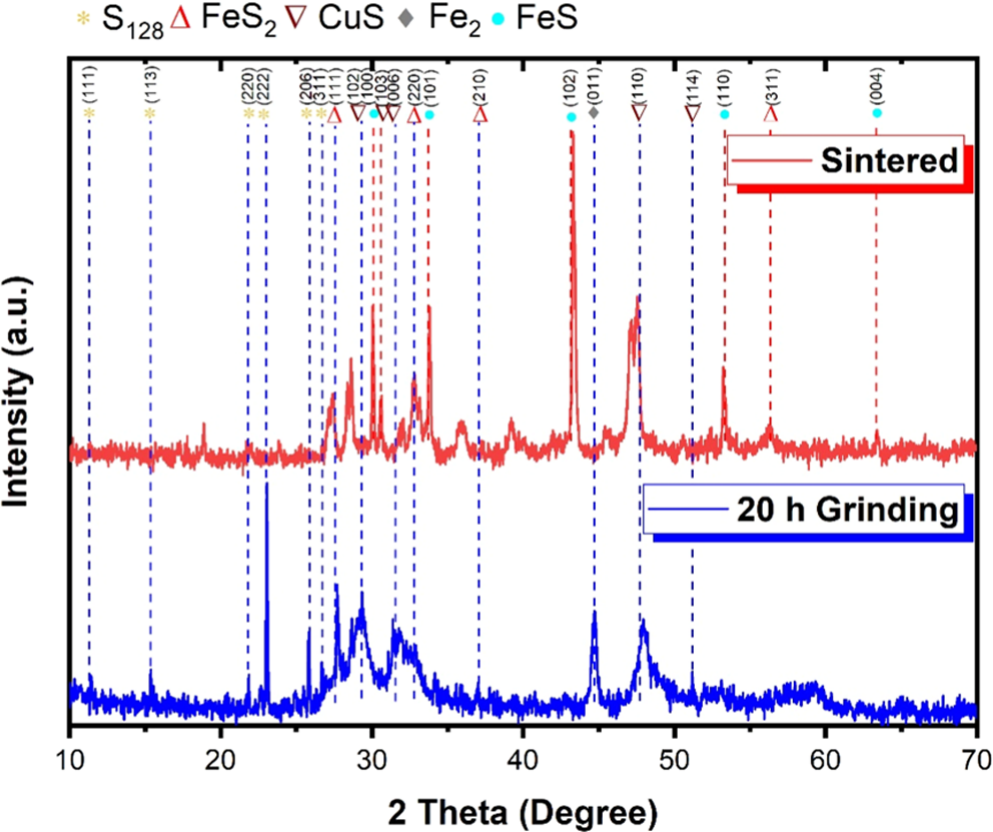
Cu–Fe–S powder after 20 h of milling in a high-energy ball mill.

A comparative study was initially conducted on the pure Cu, Fe, and S powders acquired, and the powder was manually mixed in the proportions indicated in the Materials and Methods section. X-ray Diffraction analysis was performed to characterize the material to be deposited, with the results presented in Figure 1. It is possible to observe the presence of S, Fe, and Cu in the mixture and a small amount of iron oxide already present in the Fe powder due to exposure to the atmosphere. However, the oxide proportion in the mixture is so low that the characteristic peaks are not noticeable. A prominent presence of sulfur is noted, with a main peak for the (222) plane at 2θ of 23.15° (COD 96-901-1363). Subsequently, the powder underwent 20 h high-energy milling and a sintering process. Figure 2 shows the comparative X-ray diffraction (XRD) of the powder after milling and after sintering.

According to Suryanarayana,^33^ during high-energy milling, powder particles are repeatedly flattened, cold-welded, fractured, and then welded again. The newly created surfaces allow particles to bond together; some energy is lost as heat, and a small portion is dissipated during the elastic and plastic deformations of the constituents. The severe deformation introduced into the particles results in various defects, such as dislocations, vacancies, stacking faults, and an increased number of grain boundaries, which enhance diffusivity in the material. Additionally, a slight increase in the temperature further aids the diffusion behavior, leading to the bonding of the constituent elements.

For the powder mixed in the high-energy mill, as shown in Figure 2, there is a noticeable decrease in the central sulfur peak and the disappearance of several of its crystallographic orientations compared to those of the manually mixed powder (Figure 1). Part of the sulfur combines with Cu and Fe, forming copper(II) sulfide (CuS) and iron disulfide (FeS_2_). According to Riyaz, Parveen, and Azam,^34^ the formation of CuS can be identified by the appearance of diffraction peaks for the (102), (103), (006), (110), (114), and (203) planes, which characterize the hexagonal structure of CuS (JCPDS No. 78-0876). Yuan et al.^35^ state that for iron disulfide (FeS_2_), the characteristic (stronger) diffraction peak for the (200) plane and weaker peaks for the (210) and (311) planes are observed, consistent with the standard card (JCPDS No. 42-1340). The most notable presence of iron with a characteristic peak for the (110) plane, consistent with the standard card (ICOD No. 01-089-7194), is observed in the milled powder. The probably decreased prominence of sulfur peaks provided a greater emphasis on this element in the analysis.

The absence of pure sulfur in the sintered powder was noted, which promoted an improvement during the deposition process.

The intermetallic compounds FeS, FeS_2_, and CuS, having higher dissociation and melting temperatures than those of pure sulfur, are expected to prevent sulfur evaporation, leading to a stable plasma deposition process with fewer arc openings.

In addition to the crystallographic planes mentioned previously, the formation of iron sulfide (FeS) is observed. It is characterized by a more intense peak for the (102) plane at 2θ of 43.2° and characteristic peaks for the (100), (101), (110), and (004) planes, all consistent with its standard card (ICOD 03-065-0408).

Iron disulfide requires less energy for formation, having a lower Gibbs free energy, meaning it occurs more spontaneously. Additionally, environments with higher oxygen presence favor the formation of disulfide, while environments with less oxygen or more reducing conditions promote the formation of iron sulfide.^36^ This could explain the presence of disulfide as a result of the milling process in an uncontrolled atmosphere, while sulfide appears in a reducing atmosphere and a low pressure.

Finally, in Figure 2, a comparison of the XRD patterns of the powder after 20 h of milling with the result after sintering shows the absence of pure sulfur and greater crystallinity of the material after the sintering process, with sharper and well-defined high-intensity peaks, indicating regularly and repetitively arranged atoms, which reflect X-rays more efficiently.

**Figure 3:**
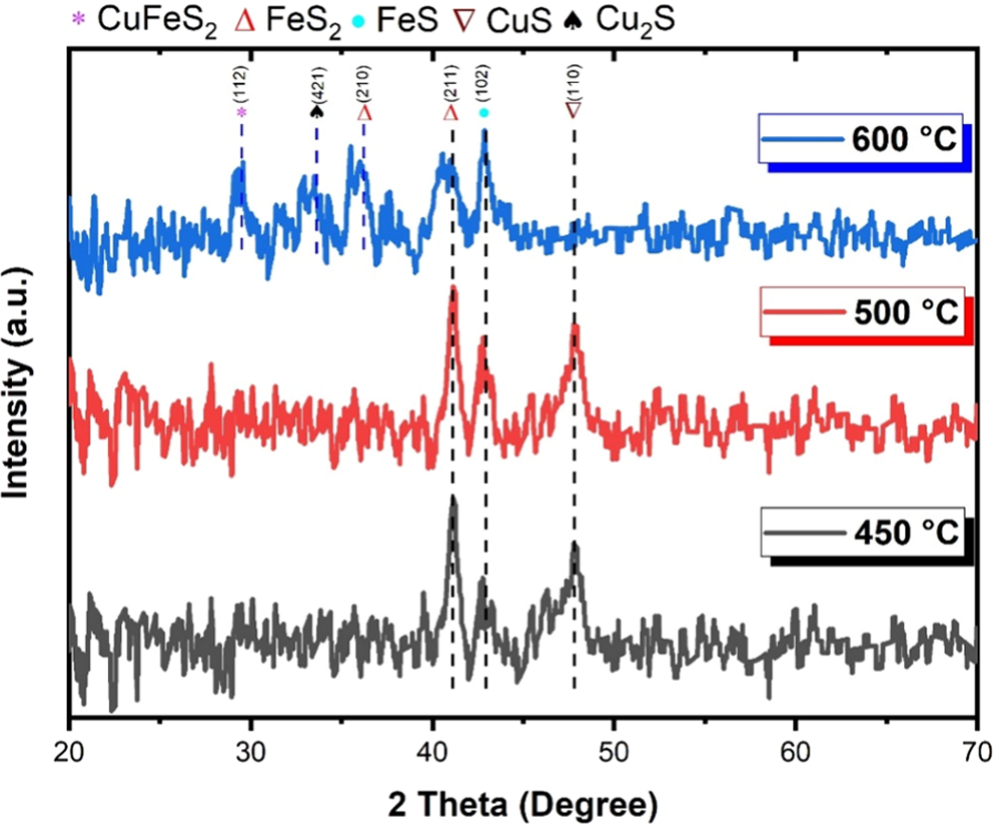
XRD of the films deposited on glass at the three temperatures.

Figure 3 illustrates the XRD results of the deposited films. According to Khalid et al.,^37^ in the sample deposited at 600 °C, peaks corresponding to chalcopyrite can be observed, as per ICDD No. 01-083-0984. The characteristic peak of FeS is also noticeable in all three samples, following the standard card (ICOD 03-065-0408).

As noted by Gao et al.,^38^ the formation of FeS_2_ can be observed for the (211) plane, consistent with the standard card (JCPDS 65-7643). For the film heated to 600 °C, the presence of the (201) plane is also noted. According to Bakr, Kamil, and Jabbar,^39^ the presence of CuS, characterized by the peak at 2θ of 47.8°, is observed, which agrees with its standard card (ICCD 06-0464) and is noted for deposition temperatures of 450 and 500 °C.

Nafees, Ikram, and Ali^40^ observed that at approximately 600 °C, copper(II) sulfide (CuS) undergoes dissociation, resulting in the formation of copper(I) sulfide (Cu_2_S) and, in oxidative atmospheres, copper oxides. The same study also found that in reducing atmospheres the formation of these oxides is significantly inhibited. Krylova, Dukštienė, and Prosyčeva^41^ identified the presence of Cu_2_S based on the characteristic peak at 2θ, observed at approximately 34°, in accordance with the standard card (JCPDS #33-490).

We can conclude that Cu, Fe, and S constituent phases are present under all three deposition conditions, but only at 600 °C was chalcopyrite of the CuFeS_2_ type found.

**Figure 4:**
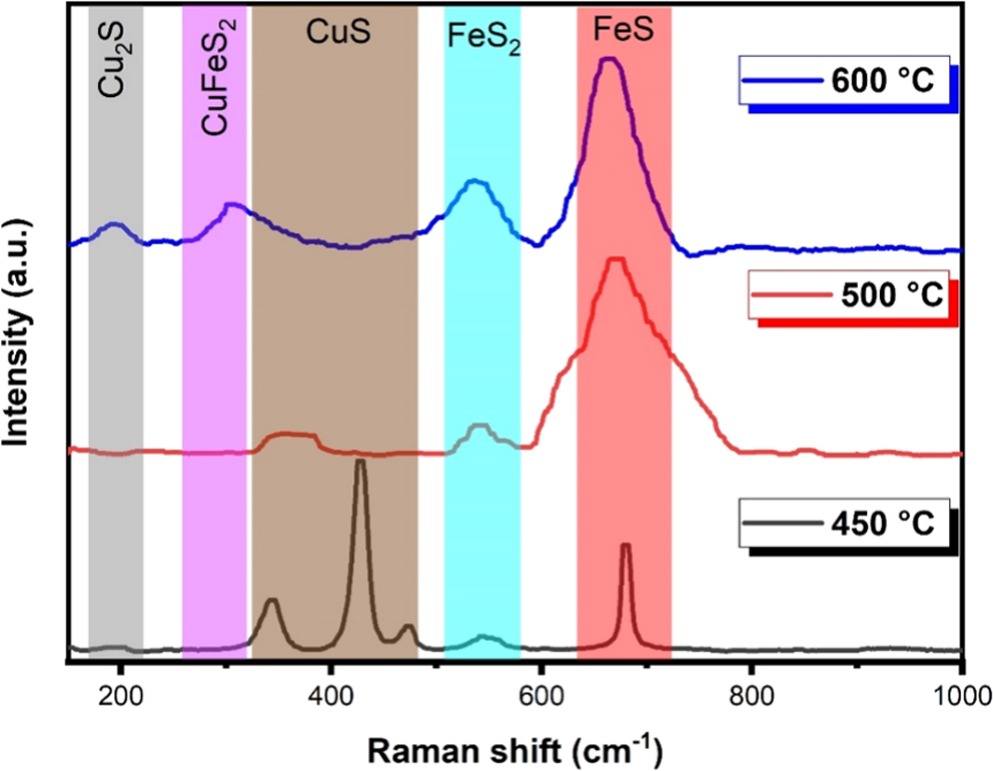
Raman spectra obtained from the three deposition temperature conditions: 450, 500, and 600 °C.

In Figure 4, we observe the presence of FeS and FeS_2_ at all deposition temperatures. According to Anthony et al. and Keller-Besrest and Collin,^42,43^ the region of vibrations highlights the presence of FeS near 700 cm^–1^ (RRUFF database at http://rruff.info/ (2024): Troilite, FeS (RRUFFID: R070242)).

According to Klimm and Botcharnikov,^44^ the presence of pyrite FeS_2_ can be noted by the disturbance near 550 cm^–1^ (RRUFF database at http://rruff.info/ (2024): Pyrite, FeS_2_ (RRUFFID: R070692)).

The Raman peak near 471 cm^–1^ corresponds to a vibration mode of CuS associated with the S–S stretching mode of S_2_ ions, indicating that Raman spectroscopy can distinguish between copper sulfides with and without S–S bonding. For this sulfide, the disturbances in lower frequency regions (below 322 cm^–1^ highlighted in the graph) can be attributed to the Cu_2–*x*_S (0.6 ≤ *x* ≤ 1) phonon mode, with the method being susceptible to the presence of this defect phase.^45,46^

According to the Raman analysis results of Jiang et al.^47^ and Minceva-Sukarova et al.,^48^ the region around 200 cm^–1^ is associated with the presence of Cu_2_S, which appears in the film deposited at 600 °C, in agreement with the XRD results.

Wang et al.^49^ state that CuFeS_2_ chalcopyrite has a crystal symmetry that can be decomposed into 15 vibrational modes. Among them, the mode around 300 cm^–1^, highlighted in Figure 4, corresponds to the nonpolar active mode with higher vibrational intensity than any other of its modes. This mode is associated with anion pairs, while the others are associated with anions and cations.

Then, films containing CuFeS_2_, FeS_2_, and Cu_2_S phases were obtained, all of which are of interest for applications in solar cells.^50−53^ However, eliminating high-purity films with secondary phases may be required for direct applications as the active layer in photovoltaic devices. Various theoretical approaches suggest effective methods, such as chemical etching, commonly used in CZTS and CIGS films.^54−56^ Furthermore, studies demonstrate that annealing, with optimized thermal treatment parameters, can eliminate unwanted phases, as exemplified by the formation of high-purity CIGSSe films without selenization.^57^ These results indicate that future investigations focused on controlling secondary phases through thermal treatment or chemical methods are promising for optimizing the properties of chalcopyrite films.

**Figure 5:**
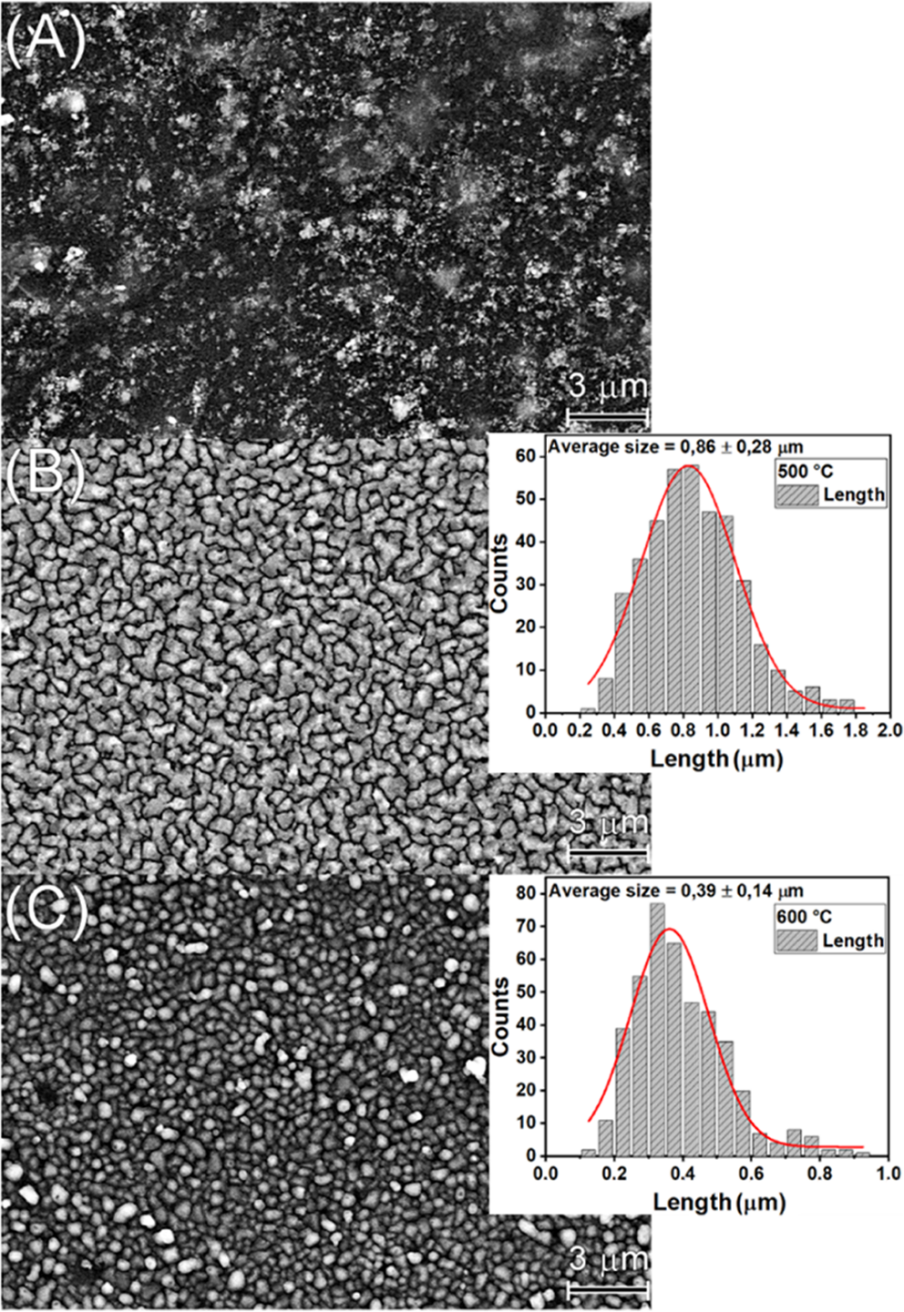
SEM-FEG for samples (A) deposited at 450 °C, (B) 500 °C, and (C) 600 °C.

Figure 5 shows the SEM images for the three deposition conditions. It is observed that for the sample deposited at 450 °C, the grains are in the nanometer scale, as indicated by the lack of resolution for grain size calculation, with low contrast that prevents complete distinction, showing only nucleation regions and low coalescence, which indicates a nonuniform film. High coalescence during film formation is observed for the films deposited at 500 and 600 °C, with micrometer-scale grains in both films.

Figure 5 also shows the grain size distribution for the samples deposited at 500 and 600 °C, obtained from SEM surface analysis using ImageJ software. Figure 5B displays a greater dispersion in grain length values, characterized by a smoother Gaussian distribution and an average grain size of 0.86 ± 0.28 μm. Figure 5C, for the sample deposited at 600 °C, presents more concentrated grain length values around 0.39 μm, with the Gaussian body indicating more homogeneous grain sizes. We can conclude that the film deposited at 600 °C is more uniformly distributed with a higher grain density.

**Figure 6:**
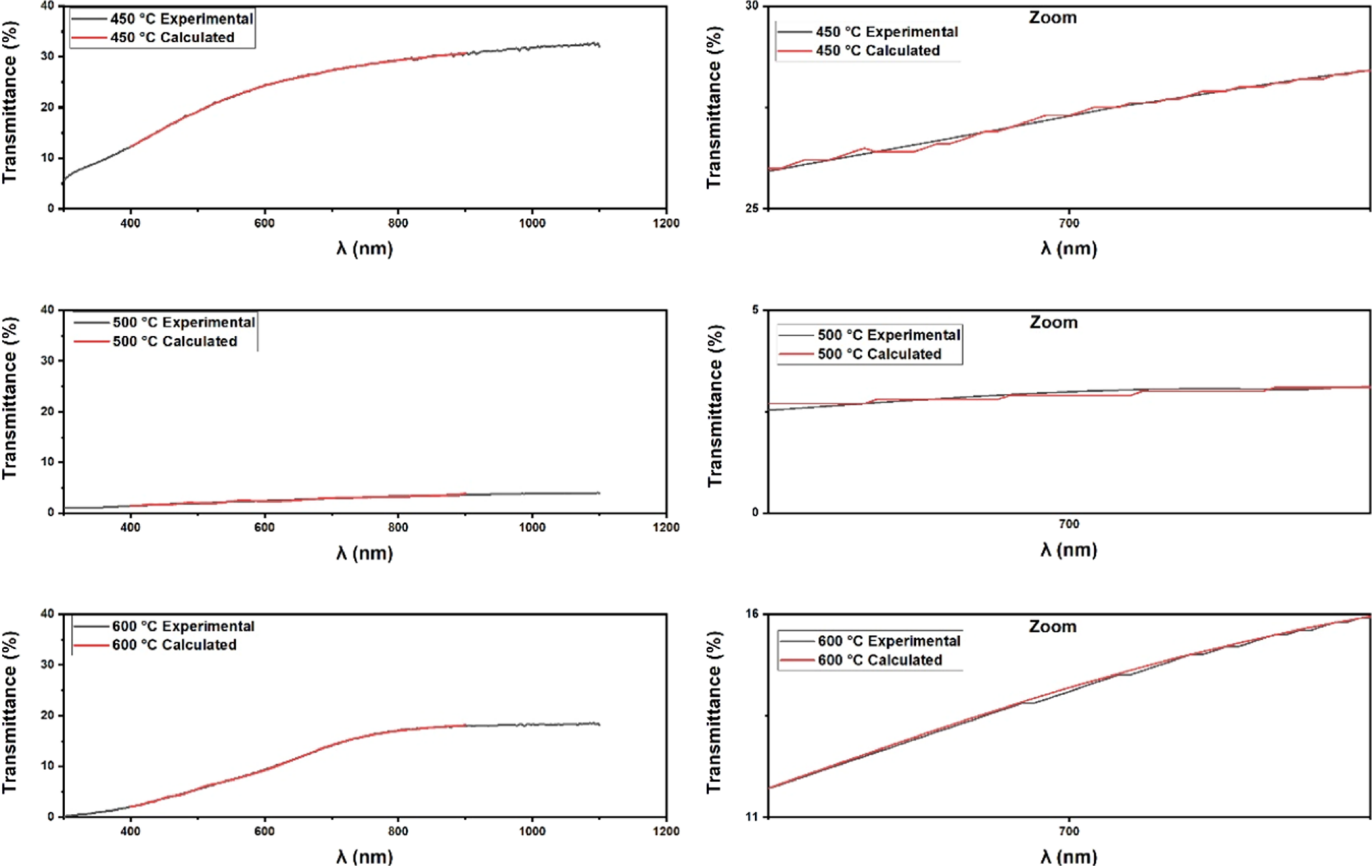
Comparison between experimental and calculated transmittances for all deposition conditions.

Figure 6 compares the actual (measured) transmittance to that obtained using the PUMA method within the wavelength range the algorithm covers.

The curves calculated by using the optical constants and thickness closely match the experimental curves. The quadratic error between the transmittance curve and the calculated curve is of a low order of magnitude for all deposition conditions, as seen in Table 2.

**Table 2:** Quadratic Error of the Difference between the Calculated Transmittance and Actual Curves for All of the Analyzed Samples

sample	450 °C	500 °C	600 °C
quadratic error	5.33 × 10^–5^	3.94 × 10^–5^	7.66 × 10^–5^

The film thicknesses obtained from the optical method can be seen in Table 3. It is noted, according to Bittencourt,^58^ that with an increase in temperature, there is a higher ionization rate in the plasma and, consequently, a higher deposition rate, leading to an increase in film thickness.

**Table 3:** Thickness of the Deposited Films

samples	thickness (nm)
450 °C	51
500 °C	124
600 °C	218

The thickness of the films deposited at 450 °C is lower than those deposited at 500 °C, which is related to the decrease in the transmittance of these films. However, this correlation does not apply to the films deposited at 600 °C, as they consist of different materials. X-ray diffraction (XRD) and Raman spectroscopy analyses indicated the presence of FeS and FeS_2_ phases in all three films. However, in the films deposited at 450 and 500 °C, CuS phases were identified, while in the films deposited at 600 °C, these phases were replaced by Cu_2_S and CuFeS_2_.

Transmittance is a function of optical parameters, including the wavelength of the incident radiation (λ), the refractive index of the substrate (*s*), the film thickness (*d*), the refractive index of the film (*n*), and the absorption coefficient of the film (*k*), according to the relation *T* = *T*(λ, *s*(λ), *d*, *n*(λ), *k*(λ)).^31^ Therefore, it is not possible to directly compare the transmittances of films with different phases by considering only the thickness as a parameter.

**Figure 7:**
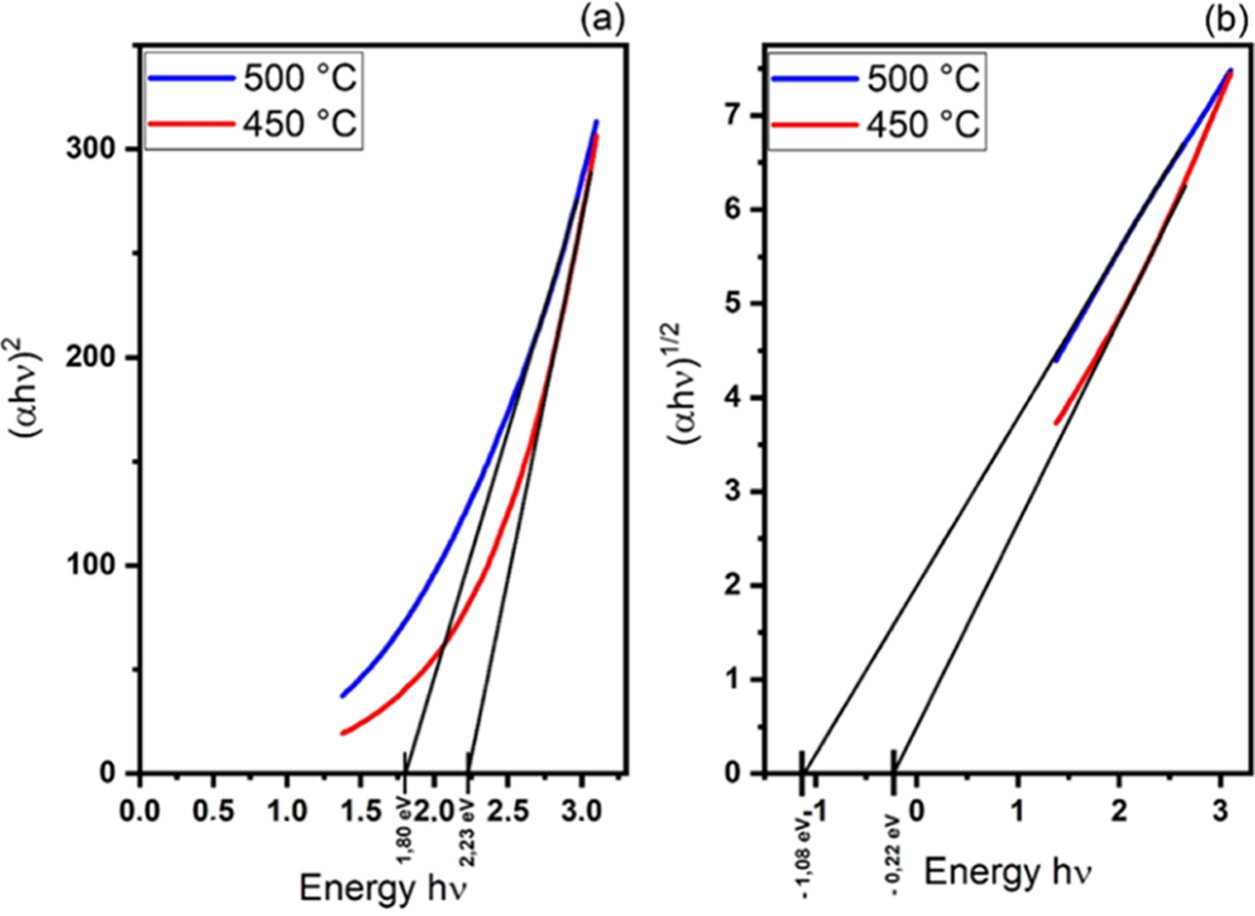
Band gap of the films deposited at 450 and 500 °C temperatures. (a) values for direct transition; (b) values for indirect transition.

In the films deposited at temperatures of 450 and 500 °C, no CuFeS_2_ chalcopyrite formation is observed; instead, only phases with Cu, Fe, and S are present. Despite being good conductors, they have direct band gaps of 2.23 and 1.80 eV for the deposition temperatures of 450 and 500 °C, respectively. This behavior can be seen in the results presented in Figure 7a.

In Figure 7b, we obtain negative results when mathematically determining the band gap values for an indirect transition, indicating that this type of transition does not exist in these films.

**Figure 8:**
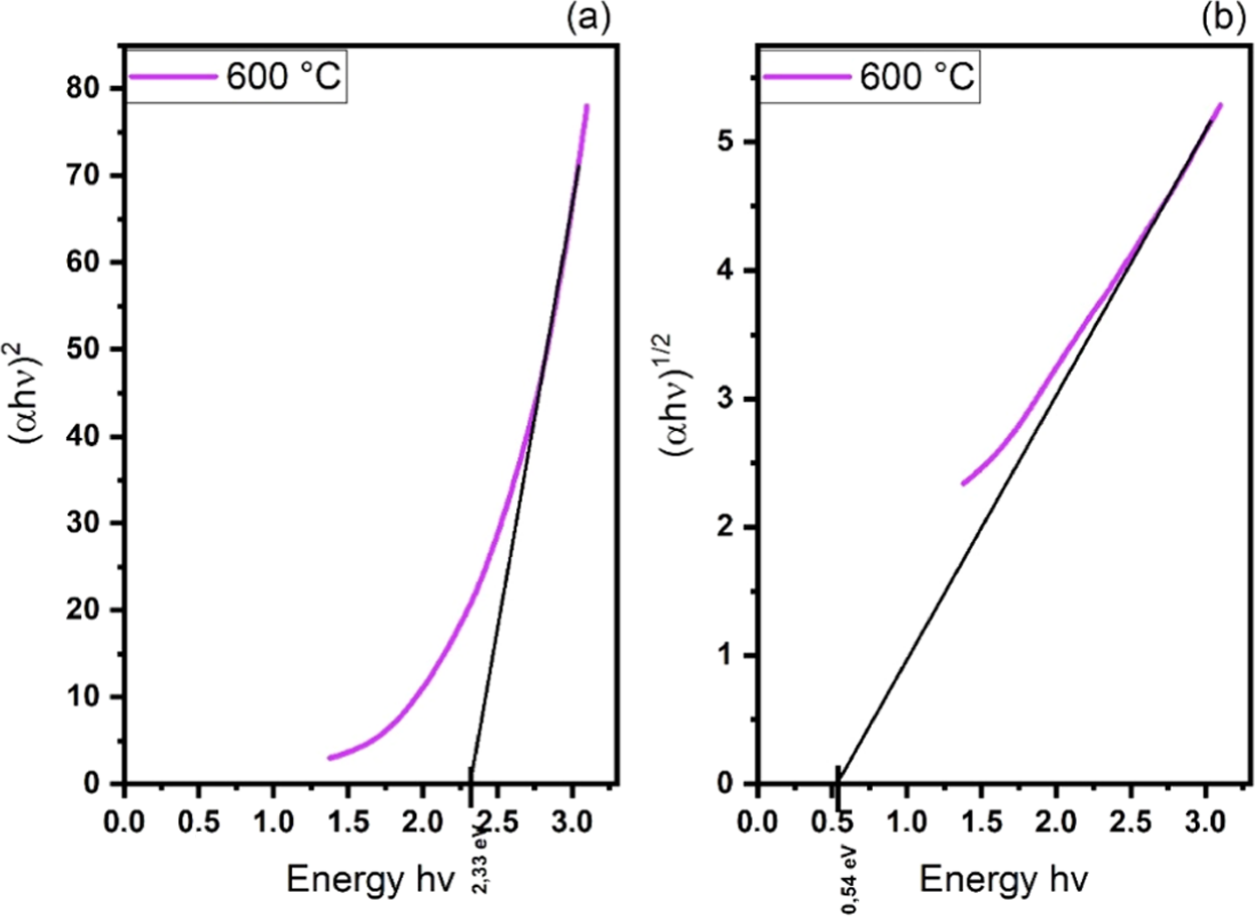
Indirect band gap obtained from CuFeS_2_ films deposited at 600 °C for (a) direct and (b) indirect transition.

Figure 8 shows that the lowest band gap result for the indirect transition was obtained for deposition at 600 °C.

The difference in band gap between the films shown in Figures 7 and 8 is because CuFeS_2_-type chalcopyrite has an indirect transition, with an intermediate band formed by the empty 3d orbitals of Fe. The band gap values obtained for this material are consistent with the literature; as for CuFeS_2_, the conduction band edge is separated from the bottom of the intermediate band by an indirect gap of approximately 0.50 eV.^9^

## Conclusions

In this study, the deposition of phase CuFeS_2_ chalcopyrite films on glass substrates was carried out by using a single deposition step through the CCyPD method. The results of this work allow us to conclude that

1. Structural studies using X-ray diffraction (XRD) and Raman spectroscopy identified the presence of CuFeS_2_ in films deposited at 600 °C, demonstrating a strong dependence between phase formation and deposition temperature.
2. The long milling process and sintering of the cylinders contribute to more stable deposition due to the higher dissociation temperature of the formed phases.
3. The CCyPD technique proves promising for thin film deposition, including chalcopyrites, which can be achieved in a single deposition step.
4. The experimentally obtained band gap values of the films agree with the theoretical values calculated and reported in the literature. This highlights the effectiveness and precision of the technique in producing high-quality films.
